# Supplementary material for: Phage engineering to overcome bacterial Tmn immunity in Dhillonvirus
Source: Commun Biol. 2025 Feb 22;8:290. doi: 10.1038/s42003-025-07730-8 (PMC11846954; doi:10.1038/s42003-025-07730-8)
Supplement: Supplementary file 1 — Supplemental material [file 42003_2025_7730_MOESM1_ESM.pdf]

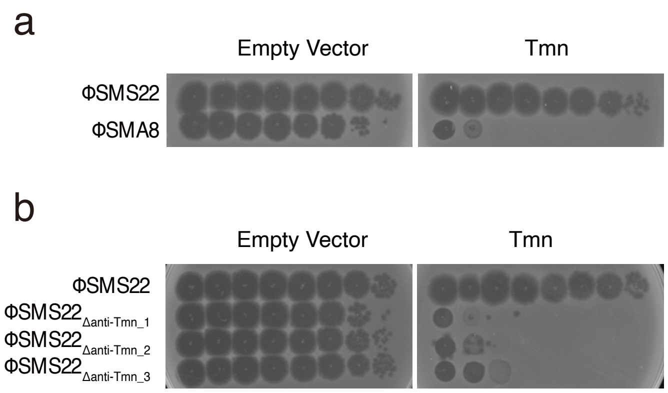

Supplementary Figure 1. Search for Tmn inhibitors (anti-Tmn). (a) Spot assay of SMS22 and SMA8 against Tmn carrying bacteria. (b) Spot assay against Tmn carrying bacteria using wild type ΦSMS22 and $\Phi SMS22_{\Delta anti\text{-}Tmn}$ with *ORF35* (*anti-Tmn*) knocked out from ΦSMS22.

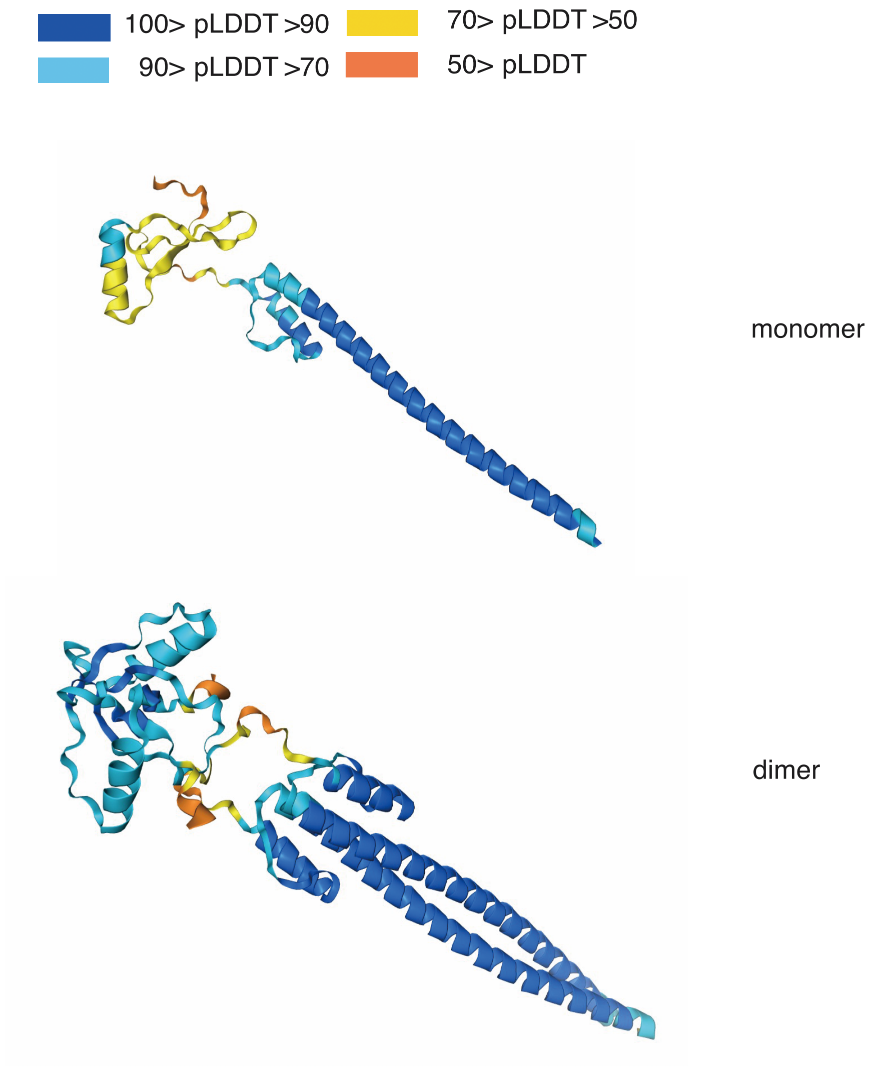

Supplementary Figure 2. Predicted structure of the monomer and dimer of anti-Tmn by AlphaFold.

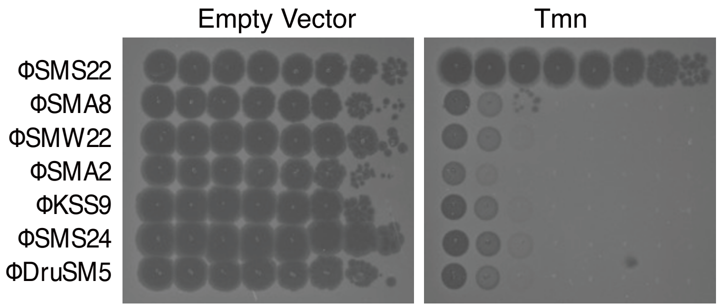

Supplementary Figure 3. Spot assay of phages ΦSMS22, ΦSMA8, ΦSMW22, ΦSMA2, ΦKSS9, ΦSMS24 and ΦDruSM5 on Tmn-carrying bacteria.

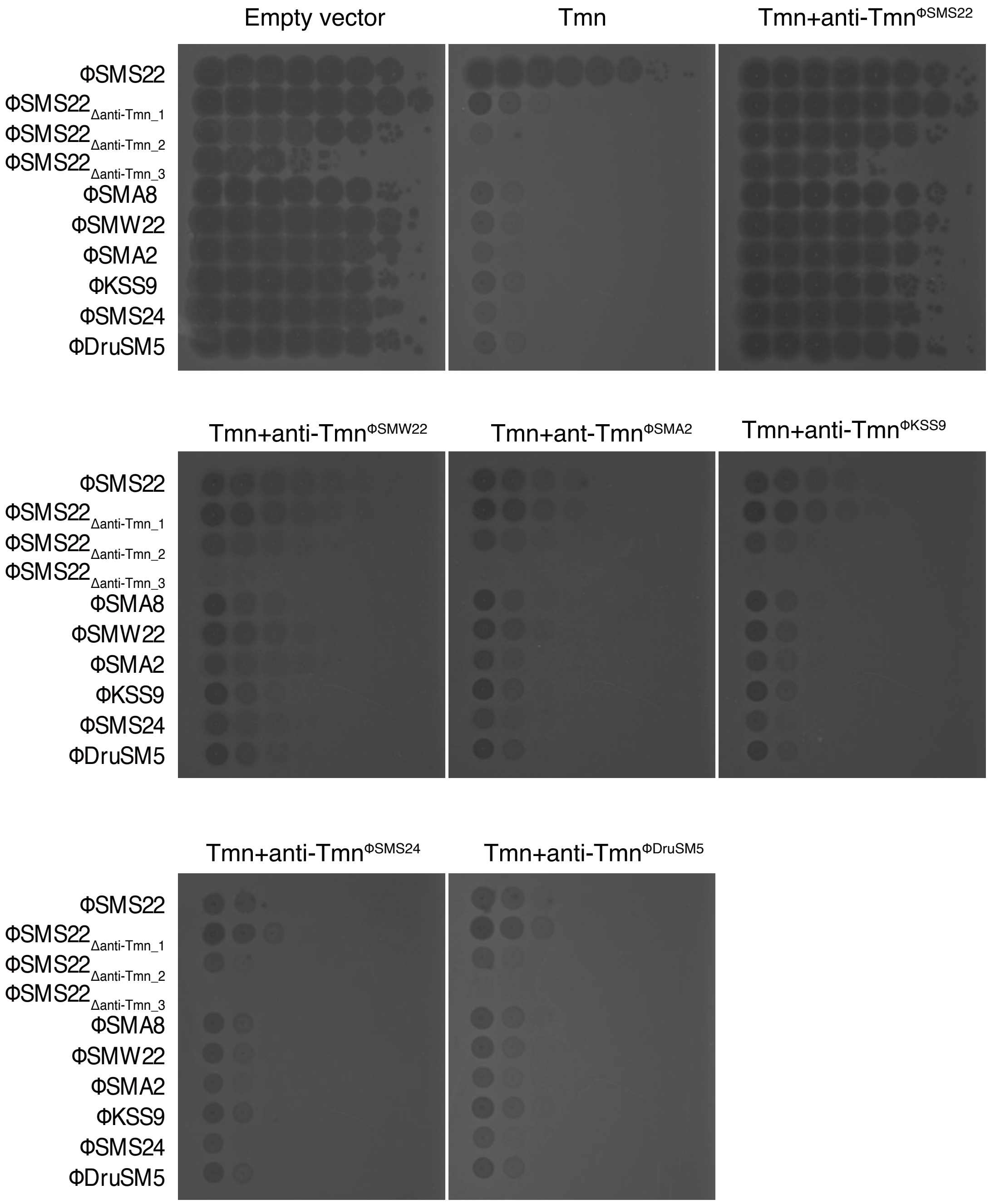

Supplementary Figure 4. Spot assay of wildtype phages (ΦSMS22, ΦSMA8, ΦSMW22, ΦSMA2, ΦKSS9, ΦSMS24, ΦDruSM5) and ΦSMS22$_{Δanti\text{-}Tmn}$ with ORF35 (anti-Tmn) knocked out from ΦSMS22 on bacteria carrying Tmn and anti-Tmn homolog.

```
ΦSMS24   MKILQEEKNYWAAQCLEAREQVERANALADTYMASYEGEEKMRKELEERFELLYELMGIM     60
ΦDruSM5  MKILQEEKNYWAAQCLEAREQVKRVSALADTYLASYEGEEKARKELEERFDLLYELMCIM     60
ΦSMS22   MKILQEEKDYWAAQCLEAREHAERVSALADTYLASYEGEEKARKELEERFDLLYELMGIM     60
ΦSMA2    MKILQEEKNYWAAQCLEAREQAERANALADTYLASYEGEEKMRKELEERLELLFELMGIM     60
ΦSMW22   MKILQEEKNYWAAQCLEAREQVERVSALADTNQALYEGEKKVRQEIEERFELLFELMGIM     60
ΦKSS9    MKILQEEKNYWAAQCLEAREQAERANALADTKQALYEGEKKVRQELEERFELLFELMGIM     60
         ********:***********:.:*..*****  * ****:* *:*:***::**:*** **

ΦSMS24   QRTGYTTTVALNRITAEGLREHCKLAEEYVRPKGSQSSIPRKGLVRIAWRA--NGSYYSD     118
ΦDruSM5  QRTGYTTTVALNRLTAEGLREHCKRAEAYVRPKGRQSSIPWKGLVHIAWRA--NGSYYSD     118
ΦSMS22   QRTGYTTTVALNRITADGLREHCKRAEEYVRPKGRQSSIARKDLVLMAWRNPTNGSFYPD     120
ΦSMA2    QRTGYTTSVALNRITADGLREHCKRAEEYVRPKGRQSKIDRKDLVLMAWRA--NGSYHSD     118
ΦSMW22   QRTGYTTSVALNRITADGLREHCKRAEEYVRPKGRRSSIDRKDLVLVAWRA--NGSYYSD     118
ΦKSS9    QRTGYTTSVALNRITAEGLREHCKRAEEYVRPKGRQSKIDRKDLVLIAWRA--NGSYYSD     118
         *******:*****:**:******* ** ****** :*.*  *.** :***   ***:: *

ΦSMS24   SRPLSRAELSRVQRVLSS--GKKTALYAIDPDYDGE     152
ΦDruSM5  SRPLSKEEMSRVQRVLSG--GKKTALYAIDPDYAEE     152
ΦSMS22   ERPLSAEESKRVARVINK--GGKTPLYAIDTDHAEE     154
ΦSMA2    CRPLSAEESKRVQRIMKT--GNKVPLYAIDPGHAEE     152
ΦSMW22   CRPLGKDEMARVQRVLTRGKGEKIALYALDPQNDGE     154
ΦKSS9    GRPLGPADMARVRRVLSSD--KKIALYAIDPDHAEE     152
          ***.  :  ** *::.     *  ***:*     *
```

Supplementary Figure 5. Protein alignment of anti-Tmn from ΦSMS22 and anti-Tmn homolog from ΦSMS22, ΦSMW22, ΦSMA2, ΦKSS9, ΦSMS24 and ΦDruSM5.

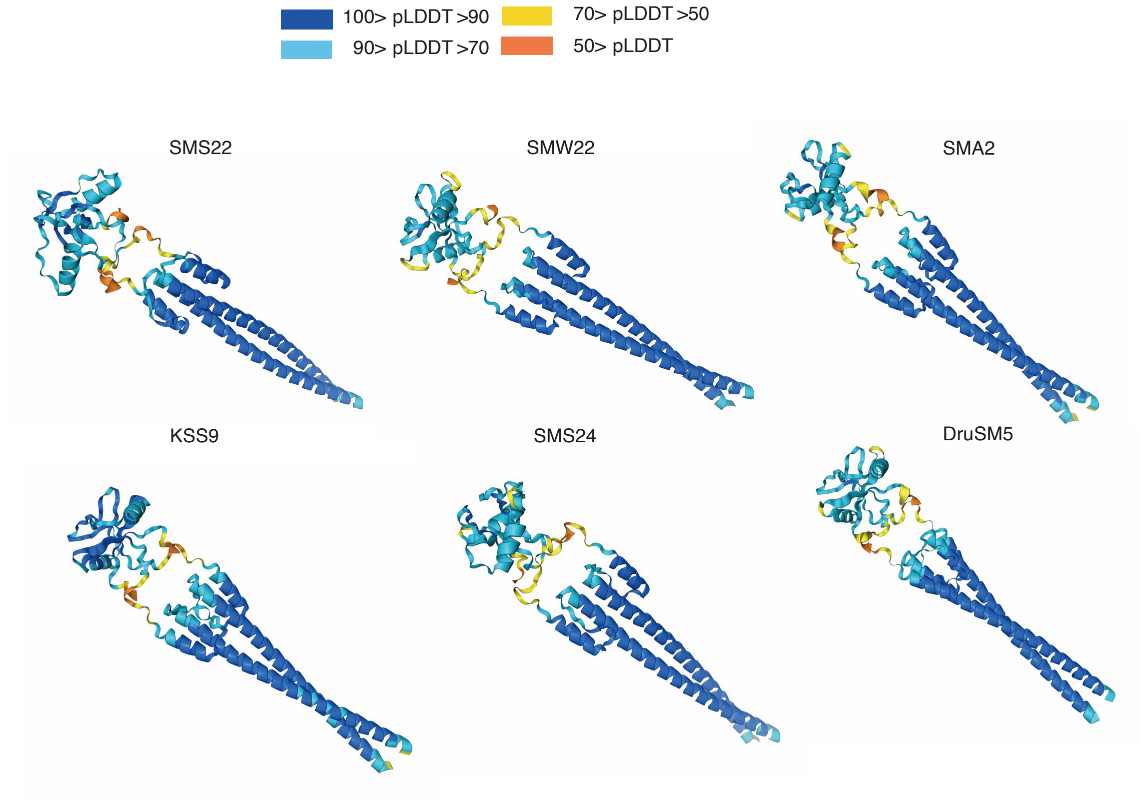

Supplementary Figure 6. Predicted structure of the dimer of anti-Tmn from ΦSMS22 and anti-Tmn homolog from ΦSMS22, ΦSMW22, ΦSMA2, ΦKSS9, ΦSMS24, ΦDruSM5 by Alphafold.

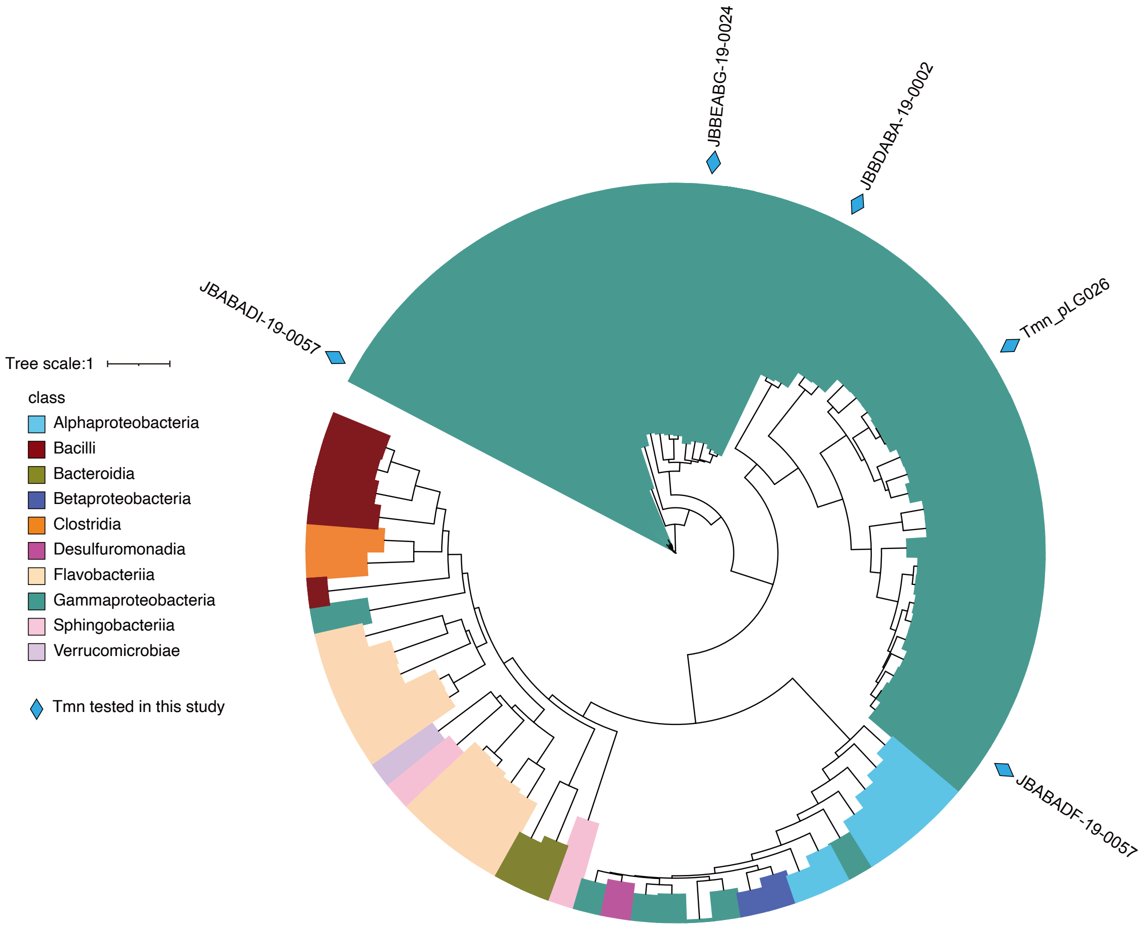

Supplementary Figure 7. Phylogenetic tree of Tmn homologs from diverse bacterial genera.

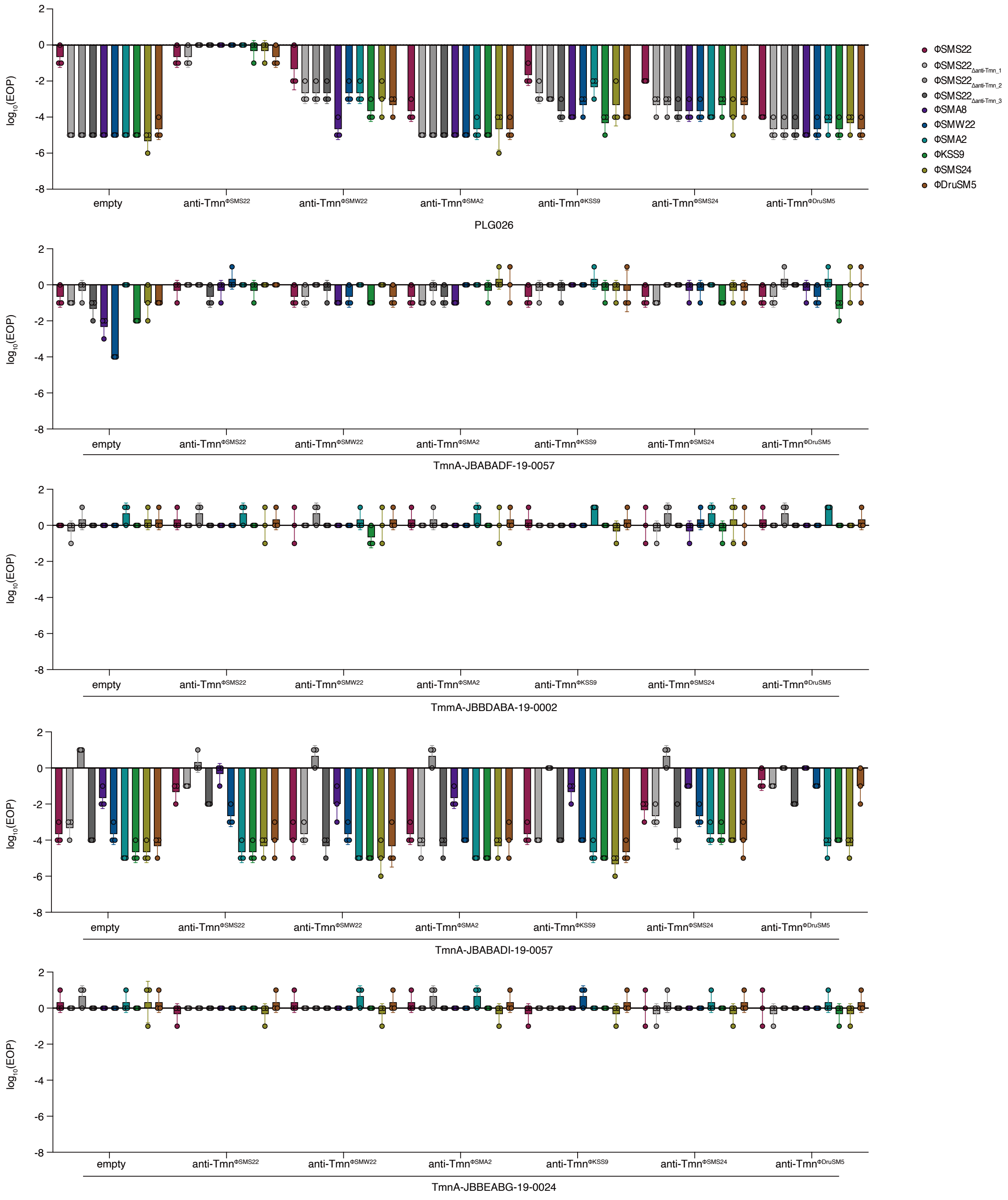

Supplementary Figure 8. Bar graph based on spot assay of wildtype phages (ΦSMS22, ΦSMA8, ΦSMW22, ΦSMA2, ΦKSS9, ΦSMS24, ΦDruSM5) and ΦSMS22$_{Δanti\text{-}Tmn}$ with *ORF35* (*anti-Tmn*) knocked out from ΦSMS22 on bacteria carrying Tmn variants and anti-Tmn homologs. The experiment was performed in triplicate, and the bar graph shows the mean values with error bars representing standard deviations.

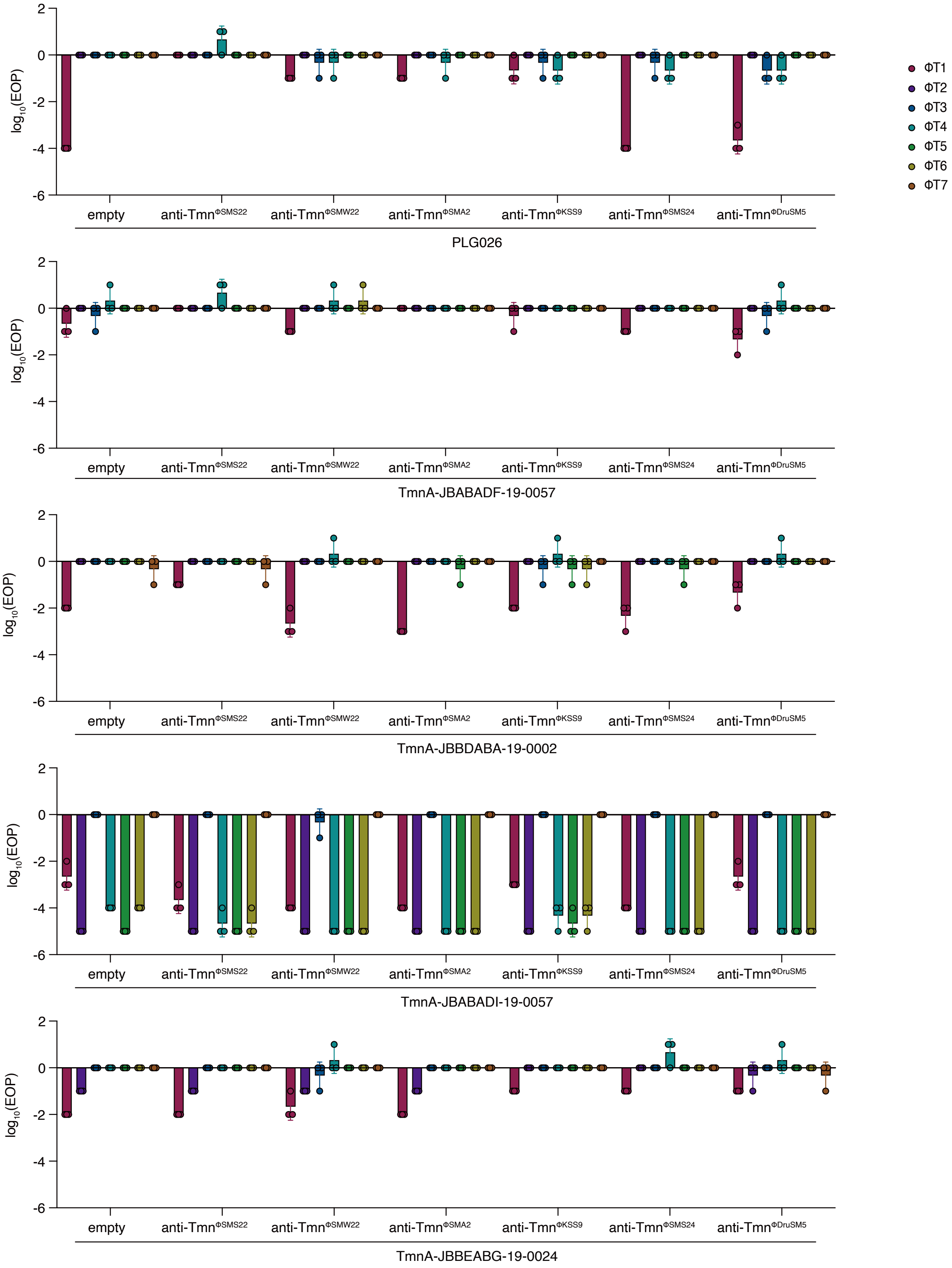

Supplementary Figure 9. Bar graph based on spot assay of ΦT1-T7 phages on bacteria carrying Tmn variants and anti-Tmn homologs. The experiment was performed in triplicate, and the bar graph shows the mean values with error bars representing standard deviations.

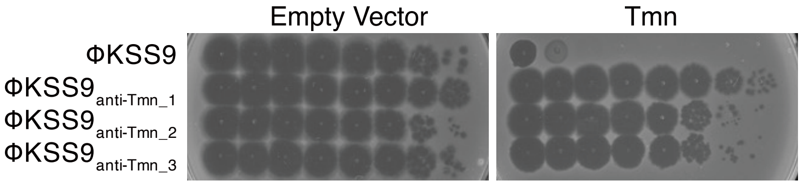

Supplementary Figure 10. Spot assay on Tmn-carrying bacteria of wild-type ΦKSS9 and $\Phi KSS9_{anti\text{-}Tmn}$ in which the *anti-Tmn* homolog of ΦKSS9 was replaced by the *anti-Tmn* of ΦSMS22.

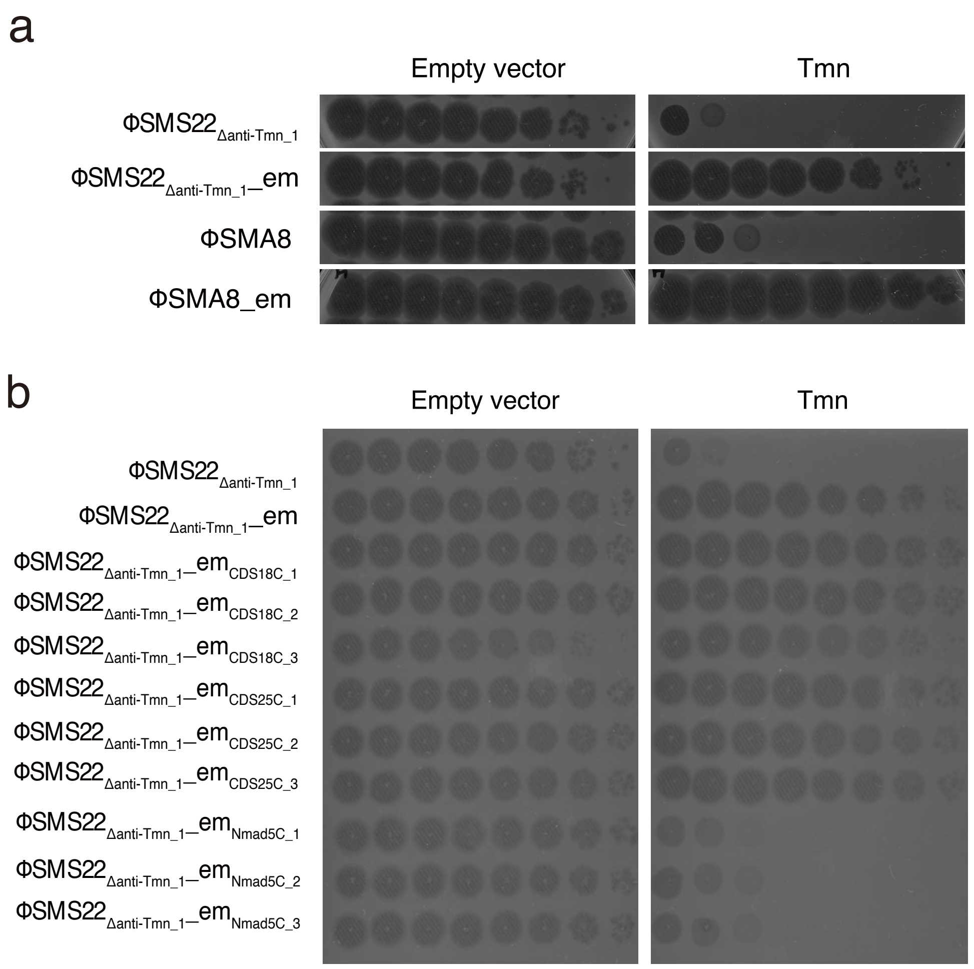

Supplementary Figure 11. Exploration of Tmn triggers. (a) Spot assay on Tmn-carrying bacteria of ΦSMS22$_{\Delta\text{anti-Tmn_1}}$ and ΦSMA8 phage and their respective mutants that escape Tmn inhibition, ΦSMS22$_{\Delta\text{anti-Tmn_1}}$_em and ΦSMA8_em. (b) Search for Tmn triggers. Spot assay against Tmn-carrying bacteria of phage with three genes mutated in ΦSMS22$_{\Delta\text{anti-Tmn_1}}$_em and reverted to WT individually.

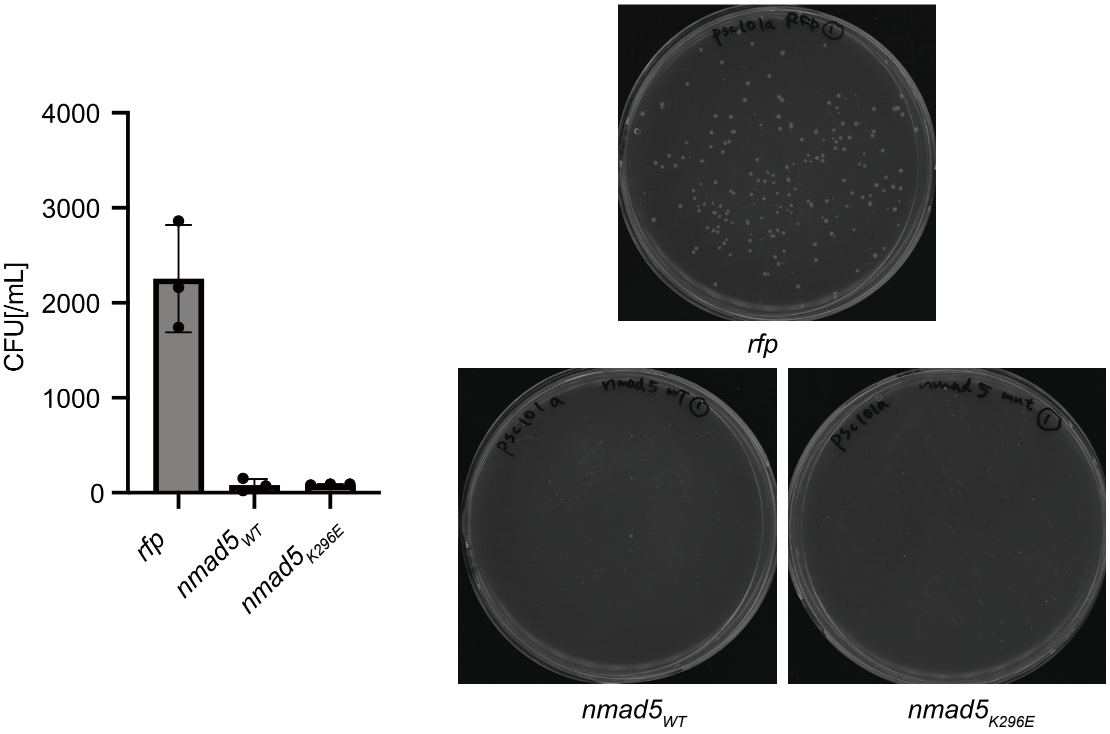

Supplementary Figure 12. Cloning of *rfp*, *nmad5*$_{\mathrm{WT}}$, and *nmad5*$_{\mathrm{K296E}}$. Due to their toxicity, the colony count after transformation with *nmad5*$_{WT}$ and *nmad5*$_{K296E}$ was lower compared to *rfp*. The experiment was performed in triplicate, and the bar graph shows the mean values with error bars representing standard deviations.

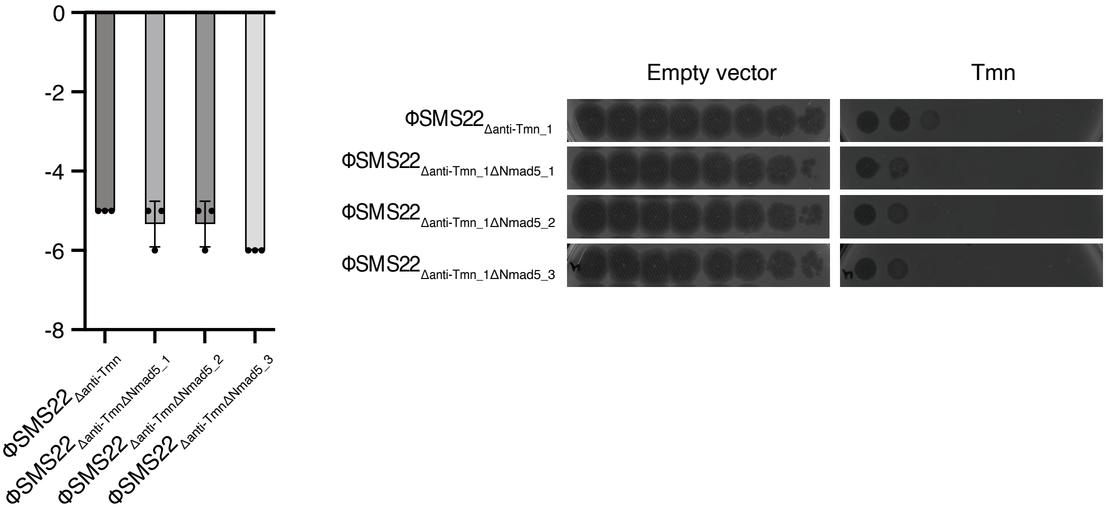

Supplementary Figure 13. Spot assay on Tmn-carrying bacteria using ΦSMS22$_{\Delta\text{anti-Tmn_1}}$ and ΦSMS22$_{\Delta\text{anti-Tmn_1}\,\Delta\text{Nmad5}}$ with the *nmad5* gene knocked out in ΦSMS22$_{\Delta\text{anti-Tmn_1}}$.

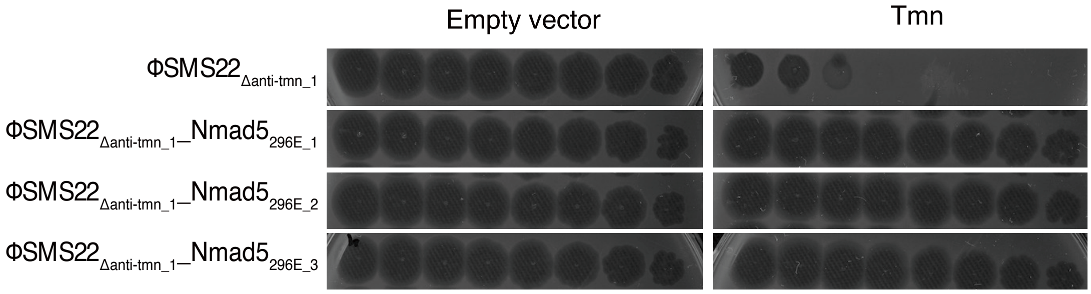

Supplementary Figure 14. Spot assay on Tmn-carrying bacteria using ΦSMS22$_{\Delta\text{anti-Tmn_1}}$ and ΦSMS22$_{\Delta\text{anti-Tmn_1}}$_Nmad5$_{296E}$ with K296E mutation of *nmad5* of ΦSMS22$_{\Delta\text{anti-Tmn_1}}$.

Supplementary Table 1. Bacterial strain list

Bacterial strain	Description	Source
DH10B	F^-, *mcrA*, Δ（*mrr-hsdRMS-mcrBC*）, Φ80d*lacZ*ΔM15, Δ*lacX74*, *deoR*, *recA*1, *araD139*, Δ(*ara* *leu*)7697, *galU*, *galK*, λ^-	Blattner FR. 2008
DH10B (PLG001)	DH10B transformed with PLG001	S. Ojima, 2024[22]
DH10B (PLG026)	DH10B transformed with PLG026	S. Ojima, 2024[22]
DH10B (PLG026+pKLC83a_anti-Tmn$^{\Phi SMS22}$)	DH10B transformed with PLG026 and pKLC83a_anti-Tmn	This study
DH10B (PLG026+pKLC83a_anti-Tmn$^{\Phi SMW22}$)	DH10B transformed with PLG026 and pKLC83a_anti-	This study
DH10B (PLG026+pKLC83a_anti-Tmn$^{\Phi SMA2}$)	DH10B transformed with PLG026 and pKLC83a_anti-Tmn$^{\Phi SMA2}$	This study
DH10B (PLG026+pKLC83a_anti-Tmn$^{\Phi KSS9}$)	DH10B transformed with PLG026 and pKLC83a_anti-Tmn$^{\Phi KSS9}$	This study
DH10B (PLG026+pKLC83a_anti-Tmn$^{\Phi SMS24}$)	DH10B transformed with PLG026 and pKLC83a_anti-	This study
DH10B (PLG026+pKLC83a anti-Tmn$^{\Phi DruSM5}$)	DH10B transformed with PLG026 and pKLC83a_anti-	This study
DH10B (PLG(TmnA-JBABADF-19-0057)+pKLC83a_anti-Tmn$^{\Phi SMS22}$)	DH10B transformed with PLG(TmnA-JBABADF-19-0057) and pKLC83a_anti-Tmn	This study
DH10B (PLG(TmnA-JBABADF-19-0057)+pKLC83a_anti-Tmn$^{\Phi SMW22}$)	DH10B transformed with PLG(TmnA-JBABADF-19-0057) and pKLC83a_anti-Tmn$^{\Phi SMW22}$	This study
DH10B (PLG(TmnA-JBABADF-19-0057)+pKLC83a_anti-Tmn$^{\Phi SMA2}$)	DH10B transformed with PLG(TmnA-JBABADF-19-0057) and pKLC83a_anti-Tmn$^{\Phi SMA2}$	This study
DH10B (PLG(TmnA-JBABADF-19-0057)+pKLC83a_anti-Tmn$^{\Phi KSS9}$)	DH10B transformed with PLG(TmnA-JBABADF-19-0057) and pKLC83a_anti-Tmn$^{\Phi KSS9}$	This study
DH10B (PLG(TmnA-JBABADF-19-0057)+pKLC83a_anti-Tmn$^{\Phi SMS24}$)	DH10B transformed with PLG(TmnA-JBABADF-19-0057) and pKLC83a_anti-Tmn$^{\Phi SMS24}$	This study
DH10B (PLG(TmnA-JBABADF-19-0057)+pKLC83a_anti-Tmn$^{\Phi DruSM5}$)	DH10B transformed with PLG(TmnA-JBABADF-19-0057) and pKLC83a_anti-Tmn$^{\Phi DruSM5}$	This study
DH10B (PLG(TmnA-JBBDABA-19-0002)+pKLC83a_anti-Tmn$^{\Phi SMS22}$)	DH10B transformed with PLG026 and pKLC83a_anti-Tmn	This study
DH10B (PLG(TmnA-JBBDABA-19-0002)+pKLC83a_anti-Tmn$^{\Phi SMW22}$)	DH10B transformed with PLG026 and pKLC83a_anti-Tmn$^{\Phi SMW22}$	This study
DH10B (PLG(TmnA-JBBDABA-19-0002)+pKLC83a_anti-Tmn$^{\Phi SMA2}$)	DH10B transformed with PLG026 and pKLC83a_anti-Tmn$^{\Phi SMA2}$	This study
DH10B (PLG(TmnA-JBBDABA-19-0002)+pKLC83a_anti-Tmn$^{\Phi KSS9}$)	DH10B transformed with PLG026 and pKLC83a_anti-Tmn$^{\Phi KSS9}$	This study
DH10B (PLG(TmnA-JBBDABA-19-0002)+pKLC83a_anti-Tmn$^{\Phi SMS24}$)	DH10B transformed with PLG026 and pKLC83a_anti-Tmn$^{\Phi SMS24}$	This study

DH10B (PLG(TmnA-JBBDABA-19-0002)+pKLC83a_anti-Tmn$^{\Phi DruSM5}$)	DH10B transformed with PLG026 and pKLC83a_anti-Tmn$^{\Phi DruSM5}$	This study
DH10B (PLG(TmnA-JBABADI-19-0057)+pKLC83a_anti-Tmn$^{\Phi SMS22}$)	DH10B transformed with PLG026 and pKLC83a_anti-Tmn	This study
DH10B (PLG(TmnA-JBABADI-19-0057)+pKLC83a_anti-Tmn$^{\Phi SMW22}$)	DH10B transformed with PLG026 and pKLC83a_anti-Tmn$^{\Phi SMW22}$	This study
DH10B (PLG(TmnA-JBABADI-19-0057)+pKLC83a_anti-Tmn$^{\Phi SMA2}$)	DH10B transformed with PLG026 and pKLC83a_anti-Tmn$^{\Phi SMA2}$	This study
DH10B (PLG(TmnA-JBABADI-19-0057)+pKLC83a_anti-Tmn$^{\Phi KSS9}$)	DH10B transformed with PLG026 and pKLC83a_anti-Tmn$^{\Phi KSS9}$	This study
DH10B (PLG(TmnA-JBABADI-19-0057)+pKLC83a_anti-Tmn$^{\Phi SMS24}$)	DH10B transformed with PLG026 and pKLC83a_anti-Tmn$^{\Phi SMS24}$	This study
DH10B (PLG(TmnA-JBABADI-19-0057)+pKLC83a_anti-Tmn$^{\Phi DruSM5}$)	DH10B transformed with PLG026 and pKLC83a_anti-Tmn$^{\Phi DruSM5}$	This study
DH10B (PLG(TmnA-JBBEABG-19-0024)+pKLC83a_anti-Tmn$^{\Phi SMS22}$)	DH10B transformed with PLG026 and pKLC83a_anti-Tmn	This study
DH10B (PLG(TmnA-JBBEABG-19-0024)+pKLC83a_anti-Tmn$^{\Phi SMW22}$)	DH10B transformed with PLG026 and pKLC83a_anti-Tmn$^{\Phi SMW22}$	This study
DH10B (PLG(TmnA-JBBEABG-19-0024)+pKLC83a_anti-Tmn$^{\Phi SMA2}$)	DH10B transformed with PLG026 and pKLC83a_anti-Tmn$^{\Phi SMA2}$	This study
DH10B (PLG(TmnA-JBBEABG-19-0024)+pKLC83a_anti-Tmn$^{\Phi KSS9}$)	DH10B transformed with PLG026 and pKLC83a_anti-Tmn$^{\Phi KSS9}$	This study
DH10B (PLG(TmnA-JBBEABG-19-0024)+pKLC83a_anti-Tmn$^{\Phi SMS24}$)	DH10B transformed with PLG026 and pKLC83a_anti-Tmn$^{\Phi SMS24}$	This study
DH10B (PLG(TmnA-JBBEABG-19-0024)+pKLC83a_anti-Tmn$^{\Phi DruSM5}$)	DH10B transformed with PLG026 and pKLC83a_anti-Tmn$^{\Phi DruSM5}$	This study

Supplementary Table 2. Primer list

Purpose	Primer name	Sequence
Exploration of Tmn-immune evasion protein Fig. 1c	SMS22$_{\Delta anti\text{-}Tmn}$_1f	CTTATATCATGGCAGAAATCAAAGTTAAGGGCCGACGCGCCAAAC
	SMS22$_{\Delta anti\text{-}Tmn}$ _1r	CAAGTCGCTGCGCGTATGGCACGTTGTTAGTAAACCAGATC
	SMS22$_{\Delta anti\text{-}Tmn}$ _2f	CTAACAACGTGCCATACGCGCAGCGACTTGAGTATG
	SMS22$_{\Delta anti\text{-}Tmn}$ _2r	GTTCTTCGGGTTGCCGTATGCTACCGCGAATGTGGAATAC
	SMS22$_{\Delta anti\text{-}Tmn}$ _3f	CACATTCGCGGTAGCATACGGCAACCCGAAGAACCACTACGAAG
	SMS22$_{\Delta anti\text{-}Tmn}$ _3r	GAGACAACACGATGGTGGTAAGACTCCACTTTATGCGATAGACACCGACCATGCAG
	SMS22$_{\Delta anti\text{-}Tmn}$ _4f	GAGTCTTACCACCATCGTGTTGTCTCCGGGGTATTAAAAAGGGCGTCCAAACCGGACAC
	SMS22$_{\Delta anti\text{-}Tmn}$ _4r	CCTGGCAATATGTTAAGGCCACCGACGAAGTTAATGGTCGAC
	SMS22$_{\Delta anti\text{-}Tmn}$ _5f	CCATTAACTTCGTCGGTGGCCTTAACATATTGCCAGGTTACG
	SMS22$_{\Delta anti\text{-}Tmn}$ _5r	CTTTGATTTCTGCCATGATATAAGCCCTTTTGTTCATATGCGCCGACCATC
	SMS22$_{\Delta anti\text{-}Tmn}$ colonyP f	GTGAAGTAACGCGGTAGCAGATG
	SMS22$_{\Delta anti\text{-}Tmn}$ colonyP r	CTTTTATGCGTAAATTGGTTGCACAATC
Variation of inhibitory activity of anti-Tmn homologs against Tmn immunity Fig. 1g	pKLC83a f	GAATTCGATCCTCTAGAGTCGAC
	pKLC83a r	CATCGGGATCCTTTCTCCTGGTAC
	SMS22 f	CATGCAGAAGAGTGAGAATTCGATCCTCTAGAGTCGACCTG
	SMS22 r	CGGGATCCTTTCTCCTGGTACCGAGCTCGAATTC
	SMW22 f	GTACCAGGAGAAAGGATCCCGATGAAAATTTTACAAGAAGAGAAAAATTACTG
	SMW22 r	GTCGACTCTAGAGGATCGAATTCTCACTCTCCGTCATTCTGC
	SMA2 f	GTACCAGGAGAAAGGATCCCGATGAAAATTTTACAAGAAGAGAAAAATTACTGGGCCGCGCAATG
	SMA2 r	GTCGACTCTAGAGGATCGAATTCTCACTCTTCAGCATGGCCTGGGTCTATC
	KSS9 f	GTACCAGGAGAAAGGATCCCGATGAAAATTTTACAAGAAGAGAAAAATTACTG
	KSS9 r	TCGACTCTAGAGGATCGAATTCTCACTCTTCTGCATGATCTGGATC
	SMS24 f	GTACCAGGAGAAAGGATCCCGATGAAAATTTTACAAGAAGAGAAAAATTACTGGGCCGCGCAATG
	SMS24 r	GTCGACTCTAGAGGATCGAATTCTCATTCTCCGTCATAGTCGGGATCTATCGCATAC
	DruSM5 f	GTACCAGGAGAAAGGATCCCGATGAAGATTTTACAAGAAGAGAAAAATTACTG
	DruSM5 r	GTCGACTCTAGAGGATCGAATTCTCACTCTTCTGCATAGTCTGGATCTATC
Synthesis of phages evading Tmn defense system using	KSS9$_{anti\text{-}Tmn}$ 1f	CTTACCATCGTTAATGACTTCCTTAACGGCATCACCTGGATC
	KSS9$_{anti\text{-}Tmn}$ 1r	CGTTCTGGATCGGCGTTCCATCCAAGTAAACAC
	KSS9$_{anti\text{-}Tmn}$ 2f	CTTGGATGGAACGCCGATCCAGAACGCTG

anti-Tmn<br>Fig. 2	KSS9$_{anti-Tmn}$ 2r	GATAGACACCGACCATGCAGAAGAGTGAGCAAAAAGATAGCTGG
	KSS9$_{anti-Tmn}$ 3f	CTTGTAAAATTTTCATTTATTTAATCTCCGTAGTGCGGATTTCCTTGCGCGGAATAAATTTGC
	KSS9$_{anti-Tmn}$ 3r	GACGAGACAATGGCACGCTTCACGCTAATCGAG
	KSS9$_{anti-Tmn}$ 4f	GATTAGCGTGAAGCGTGCCATTGTCTCGTCCTC
	KSS9$_{anti-Tmn}$ 4r	GAAGGCTTCGACAAACCAGCGGTAGAACAGGTCTTC
	KSS9$_{anti-Tmn}$ 5f	GACCTGTTCTACCGCTGGTTTGTCGAAGCCTTC
	KSS9$_{anti-Tmn}$ 5r	CAGGTGATGCCGTTAAGGAAGTCATTAACGATGGTAAGGTGTC
	anti-Tmn f	CTCACTCTTCTGCATGGTCGGTGTCTATCGCATAAAGTGGAGTCTTAC
	anti-Tmn r	GAGATTAAATAAATGAAAATTTTACAAGAAGAAAAAGATTACTGGGCCGCACAATG
	KSS9 $_{anti-Tmn}$ seqf	CTGTGTTCCGCCGTGAAGTAAC
	KSS9 $_{anti-Tmn}$ seqr	GACCGTATATGCCGAGCAAACAG
Acquisition of phages escaping Tmn immunity/Synthesis of phages evading Tmn defense system utilizing *nmad5* mutations<br>Figs 3c, 4a	SMS22 1f	CTACCGCAAGCAGATCCACGATCTAACCATC
	SMS22 1r	GTCAGTCCGATGAATTGCACGGGGAACTGTTTC
	SMS22 2f	AGTTCCCCGTGCAATTCATCGGACTGACTACTG
	SMS22 2r	GATTACTCATGCGTACCGGCCTGTTTAGCGCTAACACTATC
	SMS22 3f	GTGTTAGCGCTAAACAGGCCGGTACGCATGAGTAATCCTTG
	SMS22 3r	GTTATTGCAACGTGTCACTGGATATTTGGGCGTGGAACAACAC
	SMS22 4f	GTTCCACGCCCAAATATCCAGTGACACGTTGCAATAACAAC
	SMS22 4r	CTATTGGCATCCAGAAGATGGCAGTTACGAAGCCCAAAC
	SMS22 5f	CTTCGTAACTGCCATCTTCTGGATGCCAATAGCTGAC
	SMS22 5r	GTTAGATCGTGGATCTGCTTGCGGTAGGCTTC
Activity evaluation of Tmn variants<br>Figs. S8, S9	PLG001 EC12 f	GCGATGCCTTTCGCAATAACTAGCATAACCCCTTGGGGCC
	PLG001 EC12 r	CCCGGTGCAATGATGCGCAACGCAATTAATGTAAGTTAGC
	EC12 f	AATTGCGTTGCGCATCATTGCACCGGGTGTGTGCATATGT
	EC12 r	ATGCTAGTTATTGCGAAAGGCATCGCCACGCCACGTGCAG
	PLG001 EC26 f	GCTCAAGACTTCGCAATAACTAGCATAACCCCTTGGGGCC
	PLG001 EC26 r	ACCCATTGAGTGTTGCGCAACGCAATTAATGTAAGTTAGC
	EC26 f	GCGTTGCGCAACACTCAATGGGTATATATATCTATATGGG

	EC26 r	AGTTATTGCGAAGTCTTGAGCAATTATTTCTATAAAATAC
	PLG001 Tmn1 f	AATGCCCCCCTGTCGCAATAACTAGCATAACCCCTTGGGGCCTCTAAA
	PLG001 Tmn1 r	ATGCCTGACTTGGCTCCGTGCGCAACGCAATTAATGTAAGTTAGCTCA
	Tmn1 f	CGTTGCGCACGGAGCCAAGTCAGGCATTGTACAGCGGTGTGTTCTTTAT
	Tmn1 r	ATGCTAGTTATTGCGACAGGGGGGCATTACGCGGCTTTCTGAAAAATA
	PLG001 Tmn3 f	GTTTTCATCATGACCGCAATAACTAGCATAACCCCTTGGGGCCTCTAAA
	PLG001 Tmn3 r	AGGGCGGAAAAGCATGCGCAACGCAATTAATGTAAGTTAGCTCACTCAT
	Tmn3 f	TAATTGCGTTGCGCATGCTTTTCCGCCCTTTGGTTGGAAAAGAAAAGCG
	Tmn3 r	TGCTAGTTATTGCGGTCATGATGAAAACATATAAGAGTTTTTATTCTAT
Cloning of *nmad5* Fig. S12	psc101a f	GAATTCGATCCTCTAGAGTCGACCTG
	psc101a r	GGATCCTTTCTCCTGGTACCGAG
	RFP f	GGTACCAGGAGAAAGGATCCATGGCGAGTAGCGAAGACGTTATC
	RFP r	GACTCTAGAGGATCGAATTCTTAAGCACCGGTGGAGTGAC
	Nmad5 f	GGTACCAGGAGAAAGGATCCATGAACGTTGATTTATACCCGTCGCCATC
	Nmad5 r	GACTCTAGAGGATCGAATTCTTAGCGAGGAATGCCGCAAATCTC
Synthesis of phages of *nmad5* deletion Fig. S13	SMS22$_{\Delta Nmad5}$ _1f	GGCCCCTTTTCCGTAGTGTGTCCTCGTTGTTGGTGTGCAACCAATATATG
	SMS22$_{\Delta Nmad5}$ _5r	CAACGAGGACACACTACGGAAAAGGGGCCATTACGGCCCCTTAATTATTTG
	SMS22$_{\Delta Nmad5}$ _colonyP f	GATGATTGACGTGAAAAATATGGAATG
	SMS22$_{\Delta Nmad5}$ _colonyP r	CTTATCTTTCCAGGACTTGTCGATC

Supplementary Table 3. PCR conditions

phage	Primer 1	Primer2	template	DNA polymerase	Reaction volum	Denature temperature(℃)	Denature time(s)	Annealing temperature(℃)	Annealing time(s)	Extension temperature(℃)	Extension time(mi	Cycles
SMS22$_{\Delta\text{anti-Tmn}}$	SMS22$_{\Delta\text{anti-Tmn}}$_1f	SMS22$_{\Delta\text{anti-Tmn}}$ _1r	SMS22	KODFXNeo(TOYOBO)	50	98	10	65	30	68	10	40
	SMS22$_{\Delta\text{anti-Tmn}}$_2f	SMS22$_{\Delta\text{anti-Tmn}}$ _2r	SMS22	KODFXNeo	50	98	10	65	30	68	10	40
	SMS22$_{\Delta\text{anti-Tmn}}$_3f	SMS22$_{\Delta\text{anti-Tmn}}$ _3r	SMS22	KODFXNeo	50	98	10	65	30	68	10	40
	SMS22$_{\Delta\text{anti-Tmn}}$_4f	SMS22$_{\Delta\text{anti-Tmn}}$ _4r	SMS22	KODFXNeo	50	98	10	65	30	68	10	40
	SMS22$_{\Delta\text{anti-Tmn}}$_5f	SMS22$_{\Delta\text{anti-Tmn}}$ _5r	SMS22	KODFXNeo	50	98	10	65	30	68	10	40
ΦKSS9$_{\text{anti-Tmn}}$	KSS9$_{\text{anti-Tmn}}$ 1f	KSS9$_{\text{anti-Tmn}}$ 1r	KSS9	KODFXNeo	50	98	10	65	30	68	10	40
	KSS9$_{\text{anti-Tmn}}$ 2f	KSS9$_{\text{anti-Tmn}}$ 2r	KSS9	KODFXNeo	50	98	10	65	30	68	10	40
	KSS9$_{\text{anti-Tmn}}$ 3f	KSS9$_{\text{anti-Tmn}}$ 3r	KSS9	KODFXNeo	50	98	10	65	30	68	10	40
	KSS9$_{\text{anti-Tmn}}$ 4f	KSS9$_{\text{anti-Tmn}}$ 4r	KSS9	KODFXNeo	50	98	10	65	30	68	10	40
	KSS9$_{\text{anti-Tmn}}$ 5f	KSS9$_{\text{anti-Tmn}}$ 5r	KSS9	KODFXNeo	50	98	10	65	30	68	10	40
	anti-Tmn f	anti-Tmn r	SMS22	KODOne(TOYOBO)	50	98	10	55	5	68	5	35
ΦSMS22$_{\Delta\text{anti-Tmn_1}}$_em$_{\text{CDS18C}}$	SMS22 1f	SMS22 1r	SMS22$_{\Delta\text{anti-Tmn_1}}$	KODFXNeo	50	98	10	65	30	68	10	40
	SMS22 2f	SMS22 2r	SMS22$_{\Delta\text{anti-Tmn_1}}$_e	KODFXNeo	50	98	10	65	30	68	10	40
	SMS22 3f	SMS22 3r	SMS22$_{\Delta\text{anti-Tmn_1}}$_e	KODFXNeo	50	98	10	65	30	68	10	40
	SMS22 4f	SMS22 4r	SMS22$_{\Delta\text{anti-Tmn_1}}$_e	KODFXNeo	50	98	10	65	30	68	10	40
	SMS22 5f	SMS22 5r	SMS22$_{\Delta\text{anti-Tmn_1}}$_e	KODFXNeo	50	98	10	65	30	68	10	40
ΦSMS22$_{\Delta\text{anti-Tmn_1}}$_em$_{\text{CDS25C}}$	SMS22 1f	SMS22 1r	SMS22$_{\Delta\text{anti-Tmn_1}}$_e	KODFXNeo	50	98	10	65	30	68	10	40
	SMS22 2f	SMS22 2r	SMS22$_{\Delta\text{anti-Tmn_1}}$	KODFXNeo	50	98	10	65	30	68	10	40
	SMS22 3f	SMS22 3r	SMS22$_{\Delta\text{anti-Tmn_1}}$_e	KODFXNeo	50	98	10	65	30	68	10	40
	SMS22 4f	SMS22 4r	SMS22$_{\Delta\text{anti-Tmn_1}}$_e	KODFXNeo	50	98	10	65	30	68	10	40
	SMS22 5f	SMS22 5r	SMS22$_{\Delta\text{anti-Tmn_1}}$_e	KODFXNeo	50	98	10	65	30	68	10	40
ΦSMS22$_{\Delta\text{anti-Tmn_1}}$_em$_{\text{nmad5C}}$	SMS22 1f	SMS22 1r	SMS22$_{\Delta\text{anti-Tmn_1}}$_e	KODFXNeo	50	98	10	65	30	68	10	40
	SMS22 2f	SMS22 2r	SMS22$_{\Delta\text{anti-Tmn_1}}$_e	KODFXNeo	50	98	10	65	30	68	10	40
	SMS22 3f	SMS22 3r	SMS22$_{\Delta\text{anti-Tmn_1}}$_e	KODFXNeo	50	98	10	65	30	68	10	40
	SMS22 4f	SMS22 4r	SMS22$_{\Delta\text{anti-Tmn_1}}$_e	KODFXNeo	50	98	10	65	30	68	10	40
	SMS22 5f	SMS22 5r	SMS22$_{\Delta\text{anti-Tmn_1}}$	KODFXNeo	50	98	10	65	30	68	10	40
ΦSMS22$_{\Delta\text{anti-Tmn_1_}\Delta\text{nmad5}}$	SMS22$_{\Delta\text{nmad5}}$_1f	SMS22 1r	SMS22$_{\Delta\text{anti-Tmn_1}}$	KODFXNeo	50	98	10	65	30	68	10	40
	SMS22 2f	SMS22 2r	SMS22$_{\Delta\text{anti-Tmn_1}}$	KODFXNeo	50	98	10	65	30	68	10	40
	SMS22 3f	SMS22 3r	SMS22$_{\Delta\text{anti-Tmn_1}}$	KODFXNeo	50	98	10	65	30	68	10	40
	SMS22 4f	SMS22 4r	SMS22$_{\Delta\text{anti-Tmn_1}}$	KODFXNeo	50	98	10	65	30	68	10	40
	SMS22 5f	SMS22$_{\Delta\text{nmad5}}$ _5r	SMS22$_{\Delta\text{anti-Tmn_1}}$	KODFXNeo	50	98	10	65	30	68	10	40
ΦSMS22$_{\Delta\text{anti-Tmn}}$_nmad5$_{\text{K296E}}$	SMS22 1f	SMS22 1r	SMS22$_{\Delta\text{anti-Tmn_1}}$	KODFXNeo	50	98	10	65	30	68	10	40
	SMS22 2f	SMS22 2r	SMS22$_{\Delta\text{anti-Tmn_1}}$	KODFXNeo	50	98	10	65	30	68	10	40
	SMS22 3f	SMS22 3r	SMS22$_{\Delta\text{anti-Tmn_1}}$	KODFXNeo	50	98	10	65	30	68	10	40
	SMS22 4f	SMS22 4r	SMS22$_{\Delta\text{anti-Tmn_1}}$	KODFXNeo	50	98	10	65	30	68	10	40
	SMS22 5f	SMS22 5r	SMS22$_{\Delta\text{anti-Tmn_1}}$_e	KODFXNeo	50	98	10	65	30	68	10	40

Supplementary Table 4. Plasmid list

Purpose	Vector	Description	Source
Identification of Inhibitory Factors against Tmn Fig. 1d	PLG001	p15A ori, CmR	F. Zhang, 2020[21]
	PLG026	Tmn, p15A ori, CmR	F. Zhang, 2020[21]
Activity evaluation of anti-Tmn homolog Fig. 1g	pKLC83a	Arabinose-inducible promoter, pBR322 ori, AmpR	AH. Azam, 2023[28]
	pKLC83a_ anti-Tmn$^{\Phi SMS22}$	Arabinose-inducible promoter, anti-Tmn, pBR322 ori, AmpR	This study
	pKLC83a_ anti-Tmn$^{\Phi SMW22}$	Arabinose-inducible promoter, anti-Tmn$^{\Phi SMW22}$, pBR322 ori, AmpR	This study
	pKLC83a_ anti-Tmn$^{\Phi SMA2}$	Arabinose-inducible promoter, anti-Tmn$^{\Phi SMA2,}$ pBR322 ori, AmpR	This study
	pKLC83a_ anti-Tmn$^{\Phi KSS9}$	Arabinose-inducible promoter, anti-Tmn$^{\Phi KSS9}$, pBR322 ori, AmpR	This study
	pKLC83a_ anti-Tmn$^{\Phi SMS24}$	Arabinose-inducible promoter, anti-Tmn$^{\Phi SMS24}$, pBR322 ori, AmpR	This study
	pKLC83a_ anti-Tmn$^{\Phi DruSM5}$	Arabinose-inducible promoter, anti-Tmn$^{\Phi DruSM5,}$ pBR322 ori, AmpR	This study
Activity evaluation of Tmn variants Figs. S8, S9	PLG(TmnA-JBABADF-19-0057)	TmnA-JBABADF-19-0057, p15A ori, CmR	This study
	PLG(TmnA-JBBDABA-19-0002)	TmnA-JBABADF-19-0057, p15A ori, CmR	This study
	PLG(TmnA-JBABADI-19-0057)	TmnA-JBABADF-19-0057, p15A ori, CmR	This study
	PLG(TmnA-JBBEABG-19-0024)	TmnA-JBABADF-19-0057, p15A ori, CmR	This study
Cloning of *nmad5* Fig. S12	psc101a_RFP	Arabinose-inducible promoter, RFP, oriV, TetR	This study
	psc101a_Nmad5	Arabinose-inducible promoter, *nmad5*, oriV, TetR	This study
	psc101a_Nmad5$_{K296E}$	Arabinose-inducible promoter, *nmad5*K296E, oriV, TetR	This study
