# Supplementary material for: Phage engineering to overcome bacterial Tmn immunity in Dhillonvirus
Source: Commun Biol. 2025 Feb 22;8:290. doi: 10.1038/s42003-025-07730-8 (PMC11846954; doi:10.1038/s42003-025-07730-8)
Supplement: Supplementary file 2 — Description of Additional Supplementary Files [file 42003_2025_7730_MOESM2_ESM.docx]

Description of Additional Supplementary Files

**File name:** Supplementary Data 1

**Description:** The source data behind the figures in the paper.
